# Rehabilitation Needs of Head and Neck Cancer Patients and Stakeholders: Case Study

**DOI:** 10.3389/fonc.2021.670790

**Published:** 2021-09-24

**Authors:** Maria Karampela, Talya Porat, Vasiliki Mylonopoulou, Minna Isomursu

**Affiliations:** ^1^ Faculty of Information Technology and Electrical Engineering, University of Oulu, Oulu, Finland; ^2^ Faculty of Engineering, Dyson School of Design Engineering, Imperial College London, London, United Kingdom; ^3^ Department Of Applied Information Technology, Division of Human Computer Interaction, University of Gothenburg, Gothenburg, Sweden

**Keywords:** connected health (CH), head and neck cancer, stakeholders, informal caregivers, rehabilitation

## Abstract

**Background:**

The incidents of Head and Neck Cancer (HNC) are rising worldwide, suggesting that this type of cancer is becoming more common. The foreseen growth of incidents signifies that future rehabilitation services will have to meet the needs of a wider population.

**Objective:**

The aim of this paper is to explore the needs of patients, caregivers and healthcare professionals during HNC rehabilitation.

**Methods:**

This paper reports the empirical findings from a case study that was conducted in a cancer rehabilitation center in Copenhagen to elicit the needs of HNC cancer patients, informal caregivers and healthcare professionals.

**Results:**

Four areas of needs during the rehabilitation process were identified: service delivery, emotional, social and physical needs. Service delivery needs and emotional needs have been identified as the most prevalent.

**Conclusions:**

Stakeholders’ needs during the rehabilitation process were found to be interrelated. All stakeholders faced service delivery challenges in the form of provision and distribution of information, including responsibilities allocation between municipalities, hospitals and rehabilitation services. Emotional and social needs have been reported by HNC patients and informal caregivers, underlining the importance of inclusion of all actors in the design of future healthcare interventions. Connected Health (CH) solutions could be valuable in provision and distribution of information.

## Introduction

In 2016, a milestone has been reached and for the first time Head and Neck Cancer (HNC) was acknowledged to resemble a chronic illness (1). HNC has been recognized as one of the few cancer types that requires anticipatory treatment and prolonged rehabilitation (2). This can be related to HNC survivorship experience, which is accompanied with high morbidity and reduction in patients’ quality of life. Therefore, the main aim of rehabilitation of HNC is to improve quality of life, which is defined by the WHO “as complete physical, mental and social welfare state and not only the absence of the disease” (3). HNC cancer invades both physical and emotional aspects of life causing often facial disfigurements, disrupting the pleasure of eating (taste, smell), compromising respiratory control, sneezing and laughing mechanisms (1).

Dysphagia is the most common long-term side-effect of chemoradiotherapy (4), and is related to the difficulty of swallowing and movement of food through the ‘throat’ or pharynx (5). Dysphagia is associated among others with prolonged tube feeding and fundamental changes to eating patterns, social activities and consequently poorer quality of life (6, 7). Apart from the physical side-effects patients often experience psychological disorders. Newly diagnosed HNC patients may experience adjustment disorders and major depression (8). Emotional numbing has been reported as a factor that can increase further patients’ distress, as sharing feelings with their beloved ones was found to be a challenge on its own (9).

HNC incidence has also implications on the patient’s family and friends. Patients’ relatives and friends, known as informal caregivers, provide support to patients in the treatment and post-treatment periods (10). Caregiving is a demanding and challenging assignment. Informal caregivers of HNC patients may experience even higher levels of anxiety than patients within the six month interval following diagnosis, a state which is often related to the fear of cancer recurrence (10). Caregivers’ psychological health depends on various factors, such as patients’ disease severity, but also on their personality and resources (11). The essential role of informal caregivers and the importance of their wellbeing, has led to suggestions for specialized psychosocial interventions. These interventions have been seen as an effective approach to alleviate informal caregivers’ psychosocial burden, while also having a positive impact on the quality of patients’ lives (12).

Despite the fact that HNC patients have multiple and severe unmet needs compared to other cancer types (13) and that rehabilitation has a significant role in addressing those needs (14–16), rehabilitation is not always part of the clinical practice in HNC care (17).

**Figure 1:**
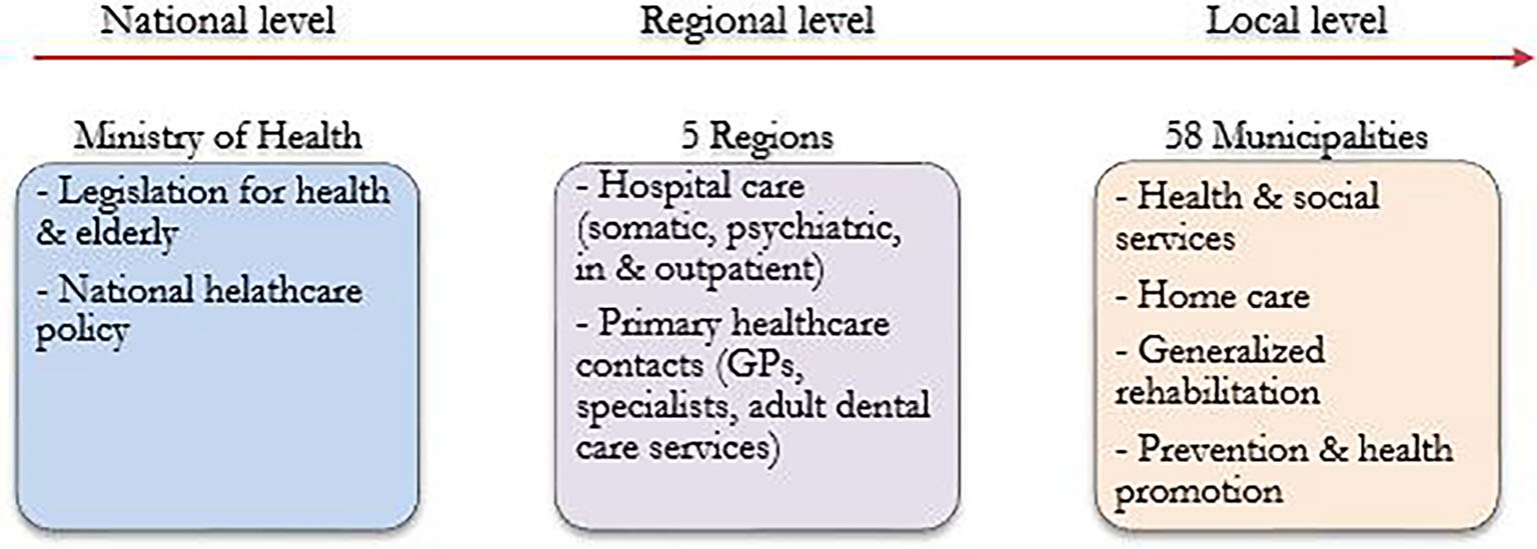
The Danish healthcare system, an overview of responsibilities between the 3 levels (National, Regional and Local) (18).

The Danish healthcare system can be characterized by decentralized administration, distributed to 5 districts and 98 municipalities. As seen in **Figure 1** below, it operates in three levels; the National (Ministry of Health), the Regional (5 regions), the Local level (98 Municipalities). The Danish healthcare may be unique in its care delivery as it allocates the rehabilitation responsibilities between Hospitals and Municipalities. However, in aspects such as healthcare quality and patient safety it is similar to other European systems (18). In addition to emergency treatments, the hospitals also provide rehabilitation services to patients with cancer, while municipalities play a key role in the prevention of the disease (19, 20). Rehabilitation of cancer patients includes a variety of interventions, such as physical therapy, psychosocial support and physical training (21–23). Cancer rehabilitation programs in Danish municipalities have been available since 2007. Municipalities are only responsible for the generalized rehabilitation interventions (24, 25), while the hospitals hold the main responsibility for specialized rehabilitation services for patients with cancer (26).

Previous studies have focused on addressing the rehabilitation needs of HNC patients and stakeholders. Healthcare professionals have reported cases in which lack of expertise to perform medical procedures and gaps in service provision led to poor services related to psychosocial needs of patients and caregivers (27, 28). The unmet information needs have been also discussed from the perspectives of patients and caregivers. Caregivers reported lack of information associated with diagnosis and treatment phases. Studies concluded that patients and caregivers also require more information about self-care, pain and distress management, while the preferable mode of information provision was from healthcare professionals and digital interventions (29). Self-management interventions have been proposed as solutions to facilitate care for patients and caregivers (30). The study of Bard et al. points out that some of the patients and caregivers’ needs were interrelated, while peer to peer social support was found to be important (30). Ringash et al. investigated the physical, emotional and cognitive needs of HNC patients and caregivers, concluding that 60% to 70% of the stakeholders reported unmet needs (31). A literature review confirmed that further investigation related to the informational and support needs of stakeholders is necessary, addressing a communication gap between patients, caregivers and healthcare professionals (32). The study of McEwen et al. utilized focus groups to address the needs of patients, caregivers and healthcare professionals, to provide insights pertinent to facilitators and barriers to recuperate functional health (17). While they found a significant amount of interrelated needs between the three stakeholders, healthcare professionals have been acknowledged to have many specific needs compared to the other two groups.

The findings above demonstrate that patients and stakeholders face a number of unmet needs in the course of HNC rehabilitation. A relation between unmet patients’ needs and caregiving burdens suggests that interventions should focus their efforts on both stakeholders (33). However, the “equation” of rehabilitation provision also includes the healthcare professionals. The present study reflects upon the studies of (17, 32, 33) by exploring the needs of patients, caregivers and healthcare professionals during HNC rehabilitation. We address the following research question: *What are the needs of the different stakeholders during Head and Neck cancer rehabilitation in the Danish context?* We explored this research question by analyzing a case study that was conducted at the Center for Kræft og Sundhed København (CKSK), during a service design course for a Master’s degree. CKSK is the largest and newest rehabilitation center in Denmark that offers rehabilitation services to approximately 1,500 cancer survivors every year. Our case study explores the phenomenon of interrelated needs and the possibility to develop Connected Health (CH) rehabilitation solutions through collaboration of HNC patients, family members and healthcare professionals.

Connected Health (CH) is a conceptual model for health management (usually *via* mobile, wireless, telehealth interventions) (34) where devices and services are designed around patient’s needs, and health data is shared, in such a way that patients are able to receive proactive and efficient care (34). CH interventions can enhance the quality of life of patients during cancer care, focusing not only on personalized solutions, but also on multidisciplinary and inclusive approaches for rehabilitation (35).

## Methods

### Case Study in CKSK Rehabilitation Center

We conducted a single-case study in the second half of 2017, where the unit of analysis was the CKSK head and neck cancer rehabilitation center. According to Yin, case study is an empirical inquiry that explores a contemporary phenomenon within its real-life context utilizing multiple sources of evidence in order to understand and explore the phenomenon under investigation (36). While most of the data collection was done in the context of Center for Kræft og Sundhed København (CKSK), supplementing data was also collected through an organization called Sundhed.dk. Sundhed.dk is a national and publicly funded health data and information portal in Denmark, complementing the service delivery needs from the viewpoint of a provider of digital solutions for rehabilitation service.

**Figure 2:**
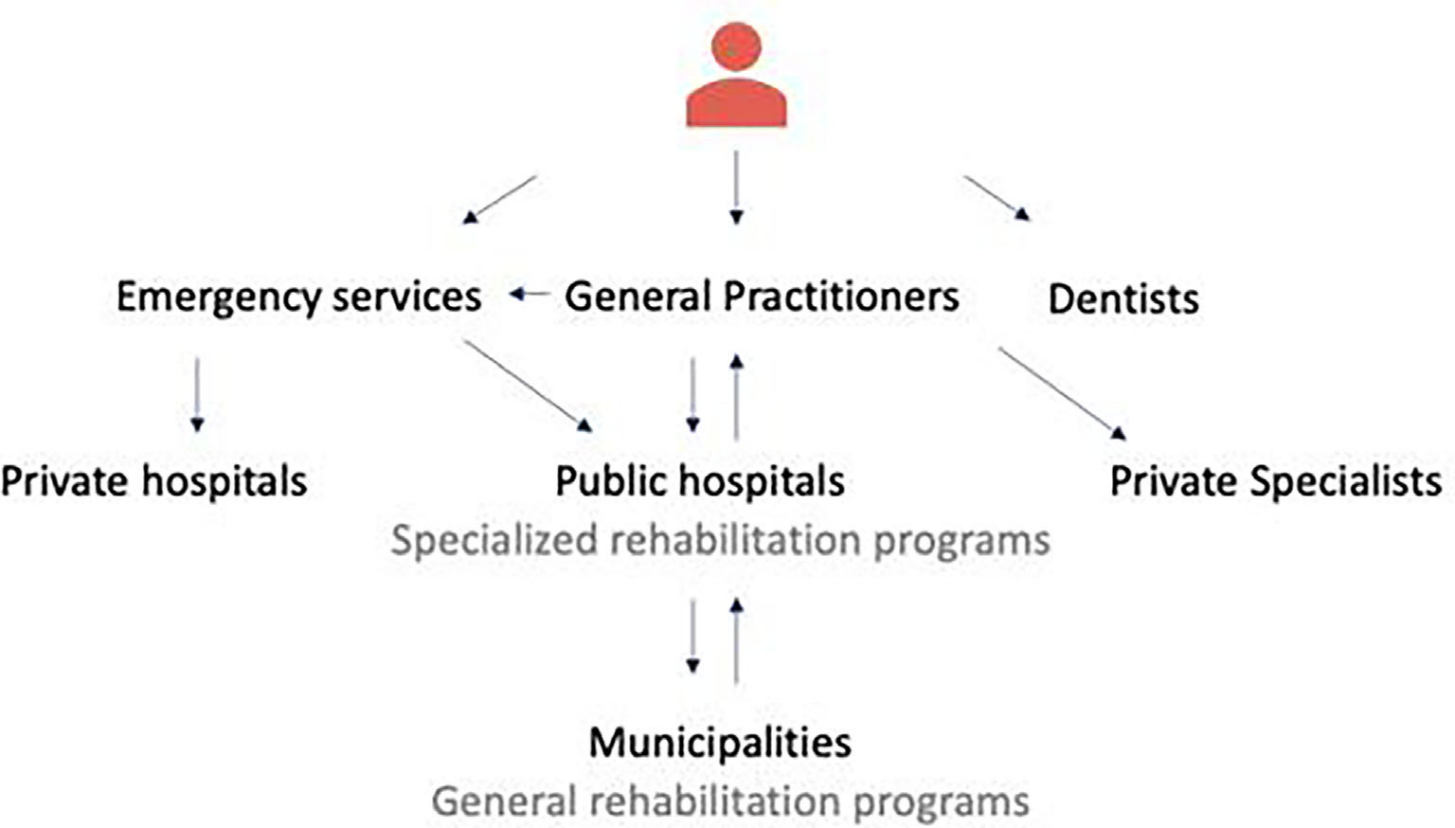
A simplified version of a cancer patient’s journey in the Danish healthcare system. The care process starts with GP’s who are the gatekeepers of the secondary care, then moves to Hospitals for provision of medical treatment and specialized rehabilitation and finally to Municipalities for generalized rehabilitation services. Dentists are self-referred specialists.

The selection of the case partners for this study reflected upon a need. Only a few municipalities in the Danish district offer rehabilitation programs for HNC patients, therefore CKSK center has to share professional expertise and knowledge with other municipalities that lack rehabilitation expertise. This problem has inspired CKSK and Sundhed.dk to collaborate in order to create a digitally assisted solution that can overcome this problem and share rehabilitation expertise and knowledge to other parts of Denmark. Before initiating the project, the case partners wanted to explore and fully comprehend the needs of the stakeholders to develop a CH solution which would serve its purpose. **Figure 2** presents a simplified version of a cancer patient’s pathway in Denmark.

### Data Collection

Data collection was performed by five groups of service design students (24 individuals), who were instructed and supervised by three service design researchers from design, engineering and information systems disciplines (one of the supervisors is a co-author of this paper). The 24 students who participated in the course were divided into five groups. Each group selected one of its members to conduct the interviews. As the goal of the case study was to collect rich, detailed information about the needs, the data collection was qualitative in nature.

The stakeholder participants were selected, as a convenience sample, by the center. The selection was based upon the following three criteria: (1) patients in their first year of rehabilitation; (2) diversity of roles of healthcare professionals; (3) willingness and availability of stakeholders to participate in relatively lengthy interviews in an active way. Data collection and understanding of the HNC domain included two phases: presentations from healthcare professionals and written material, and semi-structured interviews.

### Phase 1: Presentations From Healthcare Professionals and Written Material

Written material about HNC rehabilitation was made available to the students to understand the basic concepts, and the rehabilitation center management and nurses introduced their subjective views on problems and needs. In addition, a management representative and design professional from Sundhed.dk presented their views on possibilities and challenges of digital service delivery for cancer rehabilitation. Following this, a session with rehabilitation professionals was arranged to collect data about the needs of different areas of rehabilitation. Healthcare professionals, namely an occupational therapist, a physiotherapist, a social worker, a dietician and a nurse, gave short presentations to the groups describing their work practices and relationships to patients, as well as their specific needs in regards to their occupation. By identifying the different stakeholders and the interplay between them, the student groups were able to characterize their roles and values pertinent to the rehabilitation process (37).

### Phase 2: Semi-Structured Interviews

**Figure 3:**
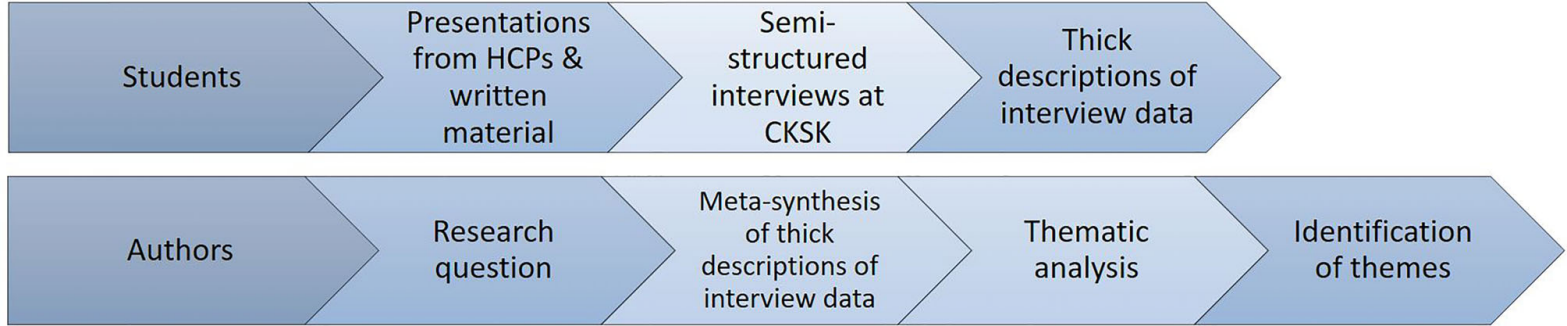
Overview of the data analysis process from the perspectives of the students and authors.

Four HNC patients, five healthcare professionals and four informal caregivers were interviewed face-to- face by all the groups of students at the premises of the center. Participants were advised to express and elaborate on their personal experiences during their rehabilitation and encouraged to comment about the content of questions, if they felt they were too personal or made them feel uncomfortable. Semi-structured interviews were organized around themes such as rehabilitation, communication, network, and close relations, with the aim to gain knowledge about the stakeholders needs and requirements. Students performed the semi-structured interviews with the different stakeholders. The interview questions were developed together with their supervisors to gain knowledge about their needs and elicit requirements for proposing future CH rehabilitation interventions. Students audio recorded the interviews and transcribed them verbatim, applying thematic analysis methods (38). Semi-structured interviews with the different stakeholders lasted between 30 minutes to 1 hour, depending on the preference of the stakeholder. All patients were interviewed in Danish (see example questions in Multimedia Appendix 1). To explore the rehabilitation services provided in municipal districts other than Copenhagen, one of the student groups conducted phone interviews with cancer coordinators from four municipalities of different sizes across Denmark. Desk research, also known as secondary research, was also used by all the groups to collect information about the relevant stakeholders and to acquire general technical and medical knowledge concerning patients with HNC (39). The students were in their final year of their master degree and were sufficiently trained to utilize the aforementioned methods as they have received specialized courses to attain this knowledge. Data collection methods were developed and implemented under the supervision of their teachers and teaching assistants. **Figure 3** presents an overview of the data analysis process from the viewpoints of the students and authors.

### Participants’ Characteristics

Four HNC patients during their rehabilitation were recruited from CKSK. Three males and one female patient, age ranged from 34 to 74, in their first year of rehabilitation provided consent to participate in this study. Patients during their first year of rehabilitation were chosen to be included, as that is the phase they have frequent interactions with the rehabilitation service. Patients with severe challenges in communication were excluded from the study. Inclusion/exclusion criteria was made by the healthcare professionals of the rehabilitation center. **Table 1** presents the patients characteristics.

**Table 1:** HNC patients’ characteristics.

ID	Participant #1	Participant #2	Participant #3	Participant #4
Gender	Male	Male	Male	Female
Age	74	67	34	56
Education^a^	BA^b^	MA^c^	MA	HD^d^
Employment^e^	No	Yes	Yes	No
Living condition	Single	With family	With family	Single
Rehab. time	9 months	12 months	3 months	10 months
HNC type	N/D^f^	N/D	Salivary glands	HPV^g^
Treatment	Surgery 33 x radiation	Surgery 35 x radiation 3 x chemotherapy	Surgery 35 x radiation	35 x radiation 2 x chemotherapy

^a^Education completed, ^b^Bachelor’s Degree, ^c^Master’s Degree, ^d^Higher Degree, ^e^Employment status, ^f^Not Defined, ^g^Human papillomavirus.

Four informal caregivers and five healthcare professionals were also recruited. The informal caregivers were three females (spouses of the patients) and one male (father of the patient). The Healthcare professionals who participated in the study were an occupational therapist, a physiotherapist, a social worker, a dietician and a nurse, who was acting as a contact person for the patients. To explore the rehabilitation services provided in municipal districts other than Copenhagen, one of the groups also conducted phone interviews with cancer coordinators from four municipalities of different sizes across Denmark.

### Data Analysis

For the purpose of this paper, a meta-synthesis of the qualitative data was conducted by the authors to abstract and generalize through the process of translation and synthesis (40, 41) - to thin out (42) - from the thick descriptions generated by the 5 groups of students (n=24). This was not a process of secondary data analysis of the primary data collected by the groups (e.g. recordings of group discussions, or transcripts of interviews), but an analysis of thick descriptions generated by the groups during the data collection process. The meta-synthesis was done through a thematic analysis, where the thick descriptions created by the data collection groups were analyzed to identify themes and sub-themes (43) that would be descriptive for identifying and understanding the needs of stakeholders. “Thick description refers to the researcher’s task of both describing and interpreting observed social action (or behavior) within its particular context” (39). Coding was used as an interpretive instrument for dealing with the content (44). We used the resulting thick description as an articulation of how we jointly see and understand the phenomenon we were studying, intertwining it with the analysis process for not merely including detail, but also interpreting and translating the detailed description of observations in the social, physical, organizational, technical and physiological context (45). All the four authors read and reviewed the thick descriptions created by the data collection groups. The authors adopted a collaborative process to analyze the thick descriptions, which was performed in two phases. Initially, the first two authors had two sessions in which the findings were discussed and themes and sub-themes were defined. In the second phase, the third and fourth authors were involved in the data analysis process to validate and discuss the justifications and choices done. After a briefing by the first author, all the authors had an additional session, in which a discussion on the themes took place and the data were grouped into the final themes and sub-themes.

### Ethical Considerations

The ethics committee of the IT-University of Copenhagen follows the principles of the Danish Code of Conduct for Research Integrity (46). An email approval for the use of data collected by the students and the analysis and interpretation work they did was asked before meta-analysis. The ethics approval process of the Center for Kræft og Sundhed København was followed in approaching and working with patients and care professionals. The participants were presented an informed consent form which they signed prior to the study, they were provided contact information of a contact person, and they were informed regarding their right to withdraw from the study at any time.

## Results

The findings below are presented as follows: (1) HNC patients’ needs, (2) Informal caregivers’ needs and (3) Healthcare professionals’ needs. The following four sub-themes have been identified: service delivery needs, emotional needs, physical needs and social needs. **Table 2** provides a summary of the results.

**Table 2:** Summary of needs - HNC patients, informal caregivers and healthcare professionals.

Service Delivery needs	Emotional needs	Social needs	Physical needs
**HNC Patients**			
Lack of information and organizational challenges Dissatisfaction caused by paper format Information management Tight rehabilitation schedule	Need for emotional support Support fuels HNC Patients motivation Need for a disease free space	Need for socializing with people they identify with Socialization fuels their motivation	Need for practical suggestions relating to nutrition and eating difficulties
**Informal Caregivers**			
Lack of information Information management	Need for support in dealing with their anxiety, stress	Need for support in dealing with their anxiety and feelings of guilt and shame	N/A
**Healthcare Professionals**			
Lack of contextual knowledge, experience and reliable information Organizational and structural challenges Need for technological support to share knowledge between municipalities	N/A	N/A	N/A^a^

^a^Not applicable.

### HNC Patients’ Needs

Patients’ rehabilitation needs are unique, as the physical and emotional symptoms are comprehensive and diverse. Four areas of needs have been identified: service delivery, emotional, social and physical.

### Service Delivery Needs

Lack of information and organizational challenges were reported by the patients. More specifically, they addressed lack of information at hospitals concerning the rehabilitation services provided by the municipalities.

A considerable ‘pain point’ for patients was related to unanswered questions. They felt that they do not have the opportunity to receive answers to questions that may suddenly arise.

The findings highlighted issues arising from the service provision. Patients were dissatisfied receiving information regarding the rehabilitation process mostly on paper:

“Yes, they could come up with something better. Something people can see on a screen, you know? That would help me, definitely.”

Another point of contention was related to information management. It was hard for patients to keep track of all the information and translate it to relevant knowledge and actions, especially during the cancer diagnosis phase:

“[ … ] it is insanely hard before the treatment period to understand what is going to happen. So really really hard. Hmm, and there was very much information overload.”

The experience of another patient concerning the management of medical information during the diagnosis period was the following:

“I could not tell them [the parents] anything because I did not have the information. I was totally far away when I was given the information.”

These findings indicate how important it is for patients to receive information in a comprehensive way in order to have an overview of their cancer treatment and rehabilitation process. The majority of them found it demanding and difficult to manage and keep control of their tight treatment schedule.

### Emotional Needs

Alongside with the physical support, patients highlighted the paramount importance of communication and psychological support from relatives, friends and healthcare professionals throughout the treatment and post-treatment periods:

“[ … ] find that person, who can coach you. It does not need to be a coach [ … ]. But find that person, who can help you to take care of your mind.”

Some of the patients underwent the rehabilitation process without having direct support from relatives or friends. They praised the experience of rehabilitation at CSCK and explained how crucial was the emotional support they received from healthcare professionals and other cancer patients.

The emotional support could trigger motivation. The need for motivation and sense of duty to participate actively in the rehabilitation process has been addressed by the HNC patients. A concrete example is pertinent to physical activities, as patients mentioned that they have to perform exercises regularly, which required a lot of discipline especially after the end of the rehabilitation program and when they were outside the center:

“My sister has completed the exercises alongside me every day when I stayed with her. It became a habit.”

They argued that the provision of emotional support is also essential as the nature of the detrimental side-effects of HNC require long-term efforts to appreciate their progress. Struggling to monitor their own progress was reported to be a demotivating factor, especially if they do not have insight into their own progress.

Finally, HNC patients expressed a demand for a disease-free space. They addressed the need to hold conversations about everyday life out of the cancer context, as they felt that they were constantly being reminded of their medical condition:

“I would rather talk about anything else [than my disease] [ … ] I had a hard time talking to my regular friends about this. Because I didn’t want to be “the sick one”. I didn’t want to talk about my cancer.”

This could pinpoint that when patients are engaging in their pre-cancer environment they desire to be again their disease-free selves.

HNC patients valued the support from relatives and family as an essential factor that had a positive impact on their life. They argued that informal caregivers supported them in coping with the burden that the disease posed and to manage their stress levels:

“My mother and wife were completely destroyed. My father also for that matter - and my brother. The whole family. It was a huge shock.”

A contact person in the rehabilitation center was considered to be a key individual. The contact person utilizes informal conversations, specialist knowledge and emotional support to customize the rehabilitation program to the patient’s specific needs:

“I’ve always been able to call her [my contact person] at any point in time - if I’ve had a bad episode - and talk to her. She has been there 100% of the time. [ … ] And if I couldn’t get a hold of her, she has always called me back [ … ].”

HNC patients asserted that the contact person was highly appreciated and acted as a motivation trigger for the patients.

### Social Needs

Apart from emotional needs, patients addressed a need for social interaction with other HNC patients. Socializing with patients that faced similar life-threatening disease considered to be essential for patients with HNC:

“[ … ] we talked during breaks at the training session, while waiting on the [workout] machines [ … ] and we talked about “what have you been operated for?” and “what have you experienced”. It was pretty comforting to get to know each other, and it encouraged me to go exercising.”

Socializing with other HNC patients enabled them to communicate their concerns pertinent to treatment and their rehabilitation challenges, including common side- effects of radiation therapy, such as sore mouth and swallowing problems. Patients argued that social support is an important part of their rehabilitation and act as a motivator to perform swallowing exercises on a regular basis:

“It has been helpful to have someone there to get me to do them [exercises]. If I had only received papers for the exercises I wouldn’t have done them.”

The demand for social support found to be higher in areas outside the capital where there is lower concentration of HNC cancer patients and lack of specialized rehabilitation centers.

Nevertheless, a few patients reported as a barrier to social support the lack of identification with other cancer patients due to issues such as age gap. A patient expressed the need to identify himself with other patients in a similar age group. Since he could not find any groups/events he felt comfortable in, he searched for videos on YouTube to learn from and identify with patients more resembling his own life situation and/or age group.

### Physical Needs

The majority of patients reported eating challenges after treatments as a side-effect of radiation therapy. Effects such as dental problems, swallowing difficulties and taste changes, along with pain and fear associated with food consumption:

“It’s not that smart, you cannot eat in public. Sometimes you swallow it down the wrong pipe. Then grab a napkin and stand and cough it up at a speed of hell. It’s not that smart to eat in public.”

Eating challenges faced by patients are typically long-term. They start from the beginning of the medical treatment, lasting long after the end of the rehabilitation period:

“[ … ] I wish it was recommended to me from the beginning, as I later learnt, that many people appreciate blueberries before and after treatment.”

### Informal Caregivers’ Needs

The needs of informal caregivers of HNC patients were pertinent to the patients’ mental health and to the practical aspects of everyday life.

### Service Delivery Needs

In terms of practical information, informal caregivers argued that there were times they felt that they missed the knowledge of how to accommodate the patients’ needs in order to establish a supportive relationship during the rehabilitation process. In addition, they experienced distress due to lack of information about the available rehabilitation options at hospital, as well as during the rehabilitation process. For example, one of the common side-effects of the radiation therapy is tooth decay, nevertheless, they argued that they were not aware that dental treatment is not part of the rehabilitation process. Another challenge that was identified related to food preparation and oral exercises. Food preparation for HNC is particularly challenging due to their new nutritional needs after the radiation therapy. Side-effects such as swallowing disorders and pain posed challenges to food preparation and required the adoption of a specific diet.

### Emotional Needs

The majority of informal caregivers often experienced stress and anxiety, which negatively affected their mental health in daily life. They experienced difficulties in expressing their feelings in the context of cancer. More specifically, relatives revealed that feelings of guilt and shame often remained unspoken, while taboo subjects such as sexual intercourse were neglected.

### Social Needs

Social needs were related to the emotional support of HNC patients. The informal caregivers expressed the desire to speak and connect with someone that understands their point of view without criticizing them:

“You’re left with forbidden feelings. Things you do not like to say out loud.”

### Healthcare Professionals’ Needs

Healthcare professionals’ needs were also acknowledged, as their input was considered valuable in having a holistic overview of the service ecology. The findings represent different healthcare professional specialties views relevant to HNC rehabilitation and were focused on service delivery needs.

### Service Delivery Needs

Healthcare professionals who were located outside the capital area stated that they lack contextual knowledge, experience and information pertinent to HNC:

“In other municipalities they need concrete knowledge about what to do with this type of patients [ … ] because they don’t have the same opportunities.”

The majority of them did not have previous experience and knowledge in treating HNC patients, indicating that these cases require specialized interventions. A desire for reliable information among healthcare professionals highlighted the potential value of interventions which are reliable, trustworthy and accurate. In some cases, healthcare professionals from municipalities in Jutland had to arrange phone consultations with CKSK to find answers to their questions:

“How do we train the musculature?”, “What kind of exercises should the physiotherapist use?”

Healthcare professionals agreed that digital solutions for knowledge sharing could have a positive impact on their performance. When healthcare professionals sought knowledge the main source of information was their colleagues. If their colleagues were not available, then they would seek knowledge through Sundhed.dk, Promedicin.dk or other sources of information. In the same vein, the value of knowledge and experience sharing between patients highlighted by healthcare professionals as a means that would facilitate their work. However, healthcare professionals mentioned that it is challenging to convince patients to gather and share their experiences in a formal setting.

While knowledge sharing is a common practice in the secondary sector, healthcare professionals highlighted that knowledge sharing practices are not common in the primary sector:

“In the secondary sector we shared a lot of data, insights and discussed a lot across different departments. In the primary care there is no such thing as knowledge sharing or discussion.”

Another finding indicates that vital treatments such as dental treatment, and rehabilitation responsibilities are allocated at various authorities on different governmental levels. A healthcare professional from a municipality in Jutland said:

“This [the dentists] we have nothing to do with. So, I believe that it is the responsibility of the hospital. We have nothing to do with that.”

This impedes the already decentralized municipal rehabilitation offer even further due to the uncertainty of distribution of responsibilities.

## Discussion

The aim of this study was to elicit the needs of stakeholders during HNC rehabilitation in a Danish context. Our findings suggest that HNC patients and stakeholders have interrelated needs. Service delivery needs have been addressed by the patients and the stakeholders as the area that posed multiple challenges. A need for emotional support has been addressed by both the patients and informal caregivers, while social needs have been reported from the perspective of patients. The main physical challenge that has been found in this study concerns dysphagia.

### Service Delivery Needs

Joint service delivery challenges have been addressed by HNC patients and stakeholders. These challenges concern unmet needs related to lack and organization of information. We found a relationship between the lack and organization of information provided for the patients in different phases of rehabilitation, and the paper format and volume of information. The provision of information in paper copies, has been seen as a factor that can lower the usability and the perceived value of information (47). Digital interactive interventions and tailor-made communication have been proposed as effective approaches for information provision to patients and informal caregivers (48–51). Digital information delivery could provide means to tackle information overload, for example, through personalization and contextualization of content. A recent multi-institutional study concluded that HNC patients prefer multiple modes of information delivery (72%), with one-to-one consultation being the most preferred method for cancer education followed by internet-based interventions (52).

Cancer patients have specific information requirements, requesting oftentimes to receive as much information as possible related to their cancer and its treatment (29, 53, 54). Information about diagnostic tests and treatment options have been reported as those areas in which patients and caregivers request to receive more information than they currently get. However, there is a lack of specialized learning resources and services to cover these ongoing needs (17, 33, 55). In line with this, our participants expressed a need for reception of supplementary or more comprehensive information in regards to swallowing exercises and dental treatments, confirming findings of other researchers (56). The provision of explanatory context and support from healthcare professionals, as well as information consistency have been valued as factors that could increase patients’ satisfaction (57). The design of information systems for HNC patients is a highly complex process underpinned not only by the complexity of the disease itself, but also from the unique requirements and needs of each patient (31, 58–61).

Another endorsed problem concerns information management from the perspective of patients and informal caregivers. They argued that managing and having an overview of information has been challenging. Information management issues, such as the difficulty to keep track of all the treatments and interventions needed during rehabilitation can be attributed to the non-digital format of information and the lack of an efficient information management service. Journeys that cancer patients undergo are often fragmented and highly individual (62). Personalization of services and patients’ involvement in the design process of interventions has been suggested as a way to identify changing needs within rehabilitation processes and to point out the specific moments that patients experience challenges (59, 63, 64). Effective communication between healthcare professionals and patients have been seen to have positive effects on patients’ health, highlighting therefore that inclusion of patients in the design of services could be beneficial in different aspects (65).

Stakeholders participation in the design of rehabilitation services could also contribute towards more efficient re-allocation of organizational and structural responsibilities. In Denmark the responsibilities are allocated at various authorities on different governmental levels, with municipalities holding the responsibility for the generalized rehabilitation interventions, while the hospitals for the specialized rehabilitation services for patients with cancer (24, 25). This organizational structure had been seen as a factor that can cause dissatisfaction, confusion and decreased the trust of patients, as it is not always transparent who holds each responsibility. The municipalities do not possess specialized proficiency needed to elaborate rehabilitation of HNC patients, due to discrepancies in the number of cancer incidences across the country (66). Knowledge and professional skills are divided across great distances, which often hinders closer collaboration across professional groups. Seamless collaboration between hospitals and municipalities could improve patients’ experience and contribute towards establishing a trustful relationship between the two parties (66).

Besides organizational and structural problems, healthcare professionals who are working in municipalities located outside the Danish capital, point out an unmet need for knowledge sharing between healthcare professionals. They proposed that peer-to-peer knowledge sharing could elicit essential and updated treatment information. Knowledge generation practices through reflection on clinical experiences, or working relationships are sources of information that healthcare professionals can benefit from, nevertheless under-utilization of sharing professional experiences and communication gaps between healthcare professionals are common practices in healthcare (17, 67). Treatment decisions in HNC are critical and require the establishment of a multidisciplinary team of healthcare professionals (68). The challenges of providing consistent speech pathology care to regional/rural patients, as well as supporting clinicians with less experience in services or with less exposure to HNC utilizing telehealth is an approach used in other countries such as Australia (69). In addition, healthcare professionals argued that knowledge input from HNC patients about their rehabilitation experience and needs, could lead towards service improvements. Based on these findings Sundhed.dk and CKSK are planning to create an information portal for patients with HNC, which will facilitate patients, informal caregivers and healthcare professionals during HNC rehabilitation.

### Emotional Needs

Emotional needs from the perspective of patients and informal caregivers include a desire for mutual psychological support. Emotional support is positively related to quality of life among cancer patients (70). Patients with deterioration in quality of life perceived a larger decrease in emotional support than patients with a positive course. HNC patients argued that emotional support enables them to continue practicing the swallowing and physical exercises. Therefore, motivation for rehabilitation and emotional support were found to have a positive relation. Similar to McEwen et al.’s findings, our HNC patients asserted also that a contact person motivated them to keep up with rehabilitation and supported them to coordinate all the interventions (17). Nevertheless, the emotional distress of newly diagnosed patients is related also to other factors such as the marital status and patients’ lifestyle (8). Half of our participants stated that they were living alone, a fact that can have a negative impact on their emotional needs. As for informal caregivers, they felt that the burden of supporting their beloved ones had an impact on their wellbeing. Literature supports that caregivers often neglect their own health as they have the notion that the patient should be the center of attention (71), therefore an increasing attention should be given to comprehend the effects this has on the mental and psychological aspects of HNC caregiving. Due to the severity of side-effects such as the ability to speak (72), HNC caregiving poses a high risk of post-traumatic stress disorders and anxiety (17, 73–75). According to Howren et al. (76), interventions for HNC caregivers is still in its infancy, therefore future healthcare interventions should be focusing on accommodating their needs.

### Social Needs

In line with previous studies, a need for social support has been also expressed by the informal caregivers and patients that participated in our study (32, 33). Informal caregivers can support and encourage patients that undergo post-treatment changes such as speech disorders, nutrition difficulties or facial features changes. Besides that, social inclusion of patients can contribute towards better functionality and better quality of life after treatment (77). As for patient-to-patient support, according to our findings, patients reported that the social support from peers had a positive impact on motivating them to continue performing physical exercises (78). HNC patients prefer to socialize with patients perceived as similar to them (30). The similarity of characteristics is based on individuals and on variables that patients perceive as relevant to their condition e.g. age, treatment, common experiences etc. (79). For example, breast cancer patients might identify themselves with everyday women, who cope with cancer rather than with the super-copers presented on television programs. These super-copers are usually famous women with cancer, who present that nothing has changed after cancer treatment (79). Moreover, the need to socialize with other patients similar to them is connected to minimization of feelings of deviance (80, 81). This also relates directly to the disease-free space the patients wish to have. Healthy people may ask more questions about a patient’s condition or even start treating them differently than before the diagnosis. This behavior change is related to various reasons such as feelings that the other person is sick and therefore in need of help, difficulties to understand the condition or fear of feeling uncomfortable. The reaction of the social environment to the person with the diagnosed condition can be a reminder of the condition to the patients.

The social aspect during rehabilitation of HNC patients has been found to be influential also for informal caregivers, as they reported negative feelings such as guilt and shame. These feelings may be common to people close to a victimized person (i.e. a person who presided as a victim by a particular situation in this case cancer). Husbands of female breast cancer patients seem to deal with their negative feelings by perceiving themselves to be better than the norm, even if that was untrue (75, 78). According to Taylor et al. (82), only 4% of female breast cancer patients were abandoned by their husbands (82). However, the husband who is leaving his sick wife was believed to be the norm by the informal caregivers (i.e. husbands). In general, comparing with people worse than one-self can lead to self-enhancement and better coping with the difficult situation (83). Psychosocial interventions have been concentrating on providing support to patients only for a limited time after the end of treatment. Nevertheless, in the cases of patients with HNC the development of long-term interventions is crucial (33, 84).

The need for social connections is in line with the Self-Determination Theory (85) arguing that ‘relatedness’ - the feeling of being understood, trusted and cared for by others, is one of the most important basic psychological needs for fostering wellbeing and enhancing motivation and sustainable behavior change.

### Physical Needs

The main physical challenge that emerged in this study concerns eating difficulties, as a side-effect of medical treatment. This should be related to the fact that all four participants of our study received radiation therapy. Interventions that may improve the problems associated with nutrition after HNC treatments are essential and have direct effects on quality of patients’ life (86). Current practice for managing dysphagia in HNC can include structured swallowing exercises usually given by a speech and language therapist prior and following cancer treatment (5, 21). In addition, mobile interventions, asynchronous telepractice applications for swallowing therapy like “SwallowIT” or screening tools for detection of swallowing, nutrition and distress status have been proposed as possible solutions to facilitate HNC patients’ needs (87–89). Strength-based exercises and range of movement exercises (maneuvers) aimed at the swallowing musculature may prevent muscle atrophy and improve prognosis for oral intake (90–92). However, patient adherence to swallowing exercises is often poor (93–95). Devices supporting the exercises have proven efficient e.g. IQoro (96), however, improved adherence may be achieved by facilitating a change in patient behavior (5) and focusing on the psychological and/or social aspects of eating and drinking and not only on the functional aspect (92, 93). A recent systematic review to identify behavioral strategies in swallowing interventions, has found that behavior change techniques that occurred more frequently in effective interventions were; practical social support, behavioral practice, self-monitoring of behavior and credible source, such as a skilled clinician delivering the intervention (5).

### Implications for Design and Future Work

The findings of this study highlight the different stakeholders’ needs in the rehabilitation process, and pave the way for different and specific CH interventions that could address some of the HNC needs. For example, based on the findings, students identified several design opportunities in the form of questions: How do we ensure that patients have the opportunity to receive answers for spontaneous questions? How might we ‘educate’ relatives to behave more naturally and not pity or feel sorry for the patient? How might we support patients to overcome the social challenges related to eating in public? How can we better prepare patients for their individual treatment and the challenges that come with it? How might we help caregivers to feel less guilty and more supported? How can we create a service that encourages patients to perform their exercises outside the center? Students also proposed initial design ideas and concepts such as developing a “digital colleague” that provides professional knowledge for healthcare professionals about HNC and creating an online community for both healthcare professionals and patients, to share their experience on HNC rehabilitation. CH interventions have the potential to support the creation of holistic, personalized and inclusive solutions to tackle the diverse and complex needs of HNC patients (35).

Based on our findings, future research should explore possibilities of CH solutions to support the psychological wellbeing of both the informal caregivers (a safe place that enables them to share their thoughts, frustrations and guilt feelings) and for patients to gain strength from people in a similar situation. In addition, digital solutions to smoothen the care pathways would be a promising area of research. Especially, the transition from cancer care to rehabilitation could be eased with digital solutions that would guide the patient through the transition. The first steps of the rehabilitation process which are characterized by overload of information could benefit from solutions that would gradually personalize the rehabilitation information and actions by integrating them into the everyday lives of the patients. The current Covid-19 outbreak poses new challenges, such as the minimization of patient-staff contact time to reduce the risk of virus transmission (97). The pandemic outbreak is an opportunity to reconsider HNC cancer management, focusing on delivering CH solutions that will support the provision of rehabilitation support from a multidisciplinary team of healthcare professionals (98, 99). For example, CH solutions for swallowing-therapy exercises utilizing video communication and sensors during HNC rehabilitation is a successful intervention used in the postCovid-19 period (35). The value of such CH rehabilitation solutions for HNC is recognized during the pandemic, paving the way to novel care delivery methods (98).

We plan to continue our collaboration with the stakeholders and take forward some of the proposed concepts and ideas. In addition, we are considering a follow-up study with HNC in the UK to evaluate if some of the proposed solutions could be relevant or adjusted to the UK population.

### Limitations

Our study has a number of limitations. The limited number of participants and the specific types of HNC and medical treatments might have introduced a bias in the findings. In addition, omitting information with regards to the patients’ surgery and treatment type (e.g., type of surgery, site of tumor or radiation dose) may have resulted in exclusion of specific patients’ rehabilitation needs. For example, a patient who underwent total laryngectomy could have different needs compared to a patient who underwent partial glossectomy. Similarly, including only patients who were capable of conducting lengthy and rigorous interviews may have excluded participants with severe communication difficulties, who may have additional needs that were not captured. However, rehabilitation needs are unique in nature and depend on a variety of factors (e.g., age, severity, treatment type, family support, socio-economic state, individual characteristics, etc). Our aim was to elicit and identify the more holistic needs that might be common across different HNC patients. The qualitative nature of this research increases the credibility of the results, as it provides in-depth content focusing on the needs of a group. One can argue that our findings represent the primary needs of stakeholders during the rehabilitation of HNC patients. In addition, the secondary synthesis of data, the multiple different data collection processes and people engaged to collect the data might have introduced bias. Further research could observe later stages of the service design process, for example observing experiences of stakeholders after the implementation of a digital intervention supporting the rehabilitation process. Despite these limitations, the study is a baseline for future endeavors, as research addressing the interrelated needs of stakeholders during rehabilitation of HNC patients is still in its early stages.

## Conclusions

While preliminary, this study offers insights related to the needs of patients and stakeholders during the HNC rehabilitation. Our findings point out that stakeholders’ needs are interrelated. All the stakeholders faced service delivery challenges contextual to lack and organization of information, as well as to information sharing and management. The distribution of responsibilities between municipalities, hospitals and rehabilitation services raised additional challenges, suggesting that reallocation of responsibilities could alleviate this issue. Interrelated emotional and social needs have been found for HNC patients and informal caregivers, underlining the importance of inclusion of all actors in the design of future healthcare interventions.

## Data Availability Statement

The original contributions presented in the study are included in the article/supplementary material, further inquiries can be directed to the corresponding author.

## Ethics Statement

Ethical review and approval was not required for the study on human participants in accordance with the local legislation and institutional requirements. Written informed consent for participation was not required for this study in accordance with the national legislation and the institutional requirements.

## Author Contributions 

All the authors have been involved in analysis and interpretation of data. MK has been responsible for drafting the first version of the manuscript and coordinating writing. TP, VM, and MI have been involved in revising the manuscript critically for important intellectual content. Portions of this manuscript were presented at the 13th EAI International Conference on Pervasive Computing Technologies for Healthcare (100). All authors contributed to the article and approved the submitted version.

## Conflict of Interest

The authors declare that the research was conducted in the absence of any commercial or financial relationships that could be construed as a potential conflict of interest.

## Publisher’s Note

All claims expressed in this article are solely those of the authors and do not necessarily represent those of their affiliated organizations, or those of the publisher, the editors and the reviewers. Any product that may be evaluated in this article, or claim that may be made by its manufacturer, is not guaranteed or endorsed by the publisher.

## Abbreviations

HNC, Head and Neck Cancer; CKSK, Center for Kræft og Sundhed København; GPs, General Practitioners.
